# Genomic analysis in patients with myxomatous mitral valve prolapse: current state of knowledge

**DOI:** 10.1186/s12872-018-0755-y

**Published:** 2018-02-27

**Authors:** S. Gasser, H. Reichenspurner, E. Girdauskas

**Affiliations:** University Heart Centre Hamburg, Department of Cardiovascular Surgery, Martinistrasse 52, 20251 Hamburg, Germany

**Keywords:** Morbus Barlow, Barlow’s disease, Bileaflet prolapse, Bileaflet mitral valve genetic analysis, Myxomatous valve disease and myxomatous mitral valve

## Abstract

**Background:**

Myxomatous mitral valve prolapse is a common cardiac abnormality. Morbus Barlow is characterized by excess myxomatous leaflet tissue, bileaflet prolapse or billowing, chordae elongation and annular dilatation with or without calcification. Extensive myxoid degeneration with destruction of the normal three-layered leaflet tissue architecture is observed histologically in such patients. Autosomal dominant inheritance with an age and sex-dependent expression has long been recognised. This review explores the current understanding of the genetics of bileaflet prolapse, with a focus on genetic analysis and the role for echocardiographical screening of the first degree relatives of affected patients.

**Methods:**

Systematic literature searches were performed using PubMed and Embase up to September 2017. In Disse et al.’s study (study one) first degree relatives of 25 patients with Morbus Barlow who underwent mitral valve repair were screened for bileaflet valve prolapse. In Nesta et al.’s study one family with three living generations of 43 individuals with 9 confirmed cases of MVP was screened. Genotyping was performed in four families for 344 microsatellite markers from Chromosome 1 to 16.

**Results:**

In study one, autosomal dominant inheritance was shown in four pedigrees. Genome-wide linkage analysis of the most informative pedigree (24 individuals, three generations) showed a significant linkage for markers mapping to chromosome 16p. Linkage to this locus was confirmed in a second family within the same study, but was excluded in the remaining two pedigrees. In study two an autosomal dominant locus was mapped to chromosome 13. 8 of the 9 individuals affected were found to suffer from bileaflet prolapse.

**Conclusions:**

Barlow’s disease is a heritable trait but the genetic causes remain largely elusive. Ch16p11.2-p12.1 is the only locus proven to be associated with bileaflet prolapse. Locus 13.q31.3-q32.1 was shown to cause bileaflet as well as posterior leaflet prolapse. This review intends to make physicians aware of genetic causes of myxomatous mitral valve prolapse, thereby emphasising the importance of cardiological examination of first-degree relatives of patients with Morbus Barlow. Integrated and more comprehensive studies are needed for identification of genes involved in this heterogenic disease. Further genomic studies may facilitate more individualised and accurate risk assessment and may help to develop possible preventive stategies for patients in the future.

## Background

Myxomatous mitral valve disease (MMVD) is a common cardiac anomaly which affects up to 5% of the general population [1]. It is characterized by a wide spectrum of clinical and electrocardiographic features, ranging from no symptoms (in the vast majority of individuals) to symptomatic mitral regurgitation, endocarditis, cerebral embolism and arrhythmias [1]. The myxomatous degeneration process often affects the whole mitral valve and therefore patients with Barlow’s disease present frequently with complex valve pathology and dysfunction, which is mostly multi-segmental. There is no current consensus in terms of the true definition of Barlow’s disease. Cardiologists diagnose Morbus Barlow when bileaflet prolapse is present, regardless of its extent. Conversely, surgeons refer to Barlow’s disease in the presence of extensive myxoid degeneration, chordae elongation and annular dilatation with prolapse of one or both mitral valve leaflets. The histological changes of myxomatous degeneration are well understood. They include accumulation of glycosaminoglycans, collagen fragmentation, disorganisation of the extracellular matrix (ECM) and increased expression of proteolytic enzymes. However, the molecular pathways leading to MMVD are yet to be clarified [2].

### Clinical presentation

MMVD is usually to be found in adults in 5th and 6th decades of life. Physicians often hear ‘murmurs’ in asymptomatic patients who are then followed for decades before being referred to a cardiologist or a cardiac surgeon. Referral is usually triggered by the development of symptoms. Typically patients with MMVD develop atrial fibrillation, shortness of breath and fatigue accompanied by left ventricular dilatation or worsening of left ventricular function. Pulmonary hypertension may be present to varying degrees. Physical examination most often shows a mid-systolic click and a mid to late systolic murmur, reflecting the timing of prolapse in the setting of excess tissue and chordal elongation [3].

### Echocardiographic findings

Echocardiography is, and may always remain, the gold standard tool for differentiation of degenerative mitral valve disease. Patients with Barlow’s disease have a characteristic echocardiographical appearance of the mitral valve apparatus. The leaflets are usually thick, bulky, elongated and distended. The chords are thickened and elongated and often ‘mesh-like’ in nature. The annulus is dilated (intercommissural distance of > 36 mm) in most cases. Prolapse is often multi-segmental. In up to 40% of patients, both leaflets are involved [4]. The insertion point of the posterior mitral leaflet (PML) is often displaced towards the left atrium, away from its normal insertion in the atrio-ventricular groove, creating a cul-de-sac at the base of the leaflet. The bodies of the bulky leaflet segments often billow above the plane of the mitral annulus. The free leaflet margins prolapse during mid to late systole, in the setting of chordal elongation, or in early systole if chordal rupture has already occurred. Calcification of the annulus and of papillary muscle tips may be present. In the late systole “papillary muscle traction” causes the papillary muscle tip to be drawn upward to the plane of the mitral annulus instead of downward towards the apex, as is the normal motion [5]. This may be demonstrated echocardiographically as a left ventricular wall motion abnormality [6].

### Histological findings

The degenerative process within the mitral valve has two distinct histological phenotypes: diffuse myxomatous degeneration (MMVD or so-called ‘Barlow’s’ disease) and fibroelastic deficiency (FED) which is characterized by thin, translucent leaflets without excessive leaflet tissue. The pathological features of FED consist of both qualitative and quantitative abnormalities in the connective tissue structure. This includes deficiencies of collagen, elastin and proteoglycans. The histological hallmark of myxomatous mitral valve degeneration is myxoid tissue infiltration resulting in destruction of the three-layered leaflet architecture. Moreover, histological examination reveals often-severe alterations in collagen structure [7].

### History of myxomatous mitral valve disease

Barlow initially described the syndrome characterised by a late systolic murmur and non-ejection systolic click. He and his colleagues performed cardiac catheterisation and phonocardiography, confirming that these auscultatory findings were due to mitral regurgitation occurring in late systole. They emphasized that the condition they were reporting was distinct from apical pan-systolic murmurs with late systolic accentuation as a result of mitral regurgitation [5]. In 1964, Segal and Likoff reported late systolic “bulging” of the anterior mitral leaflet associated with the onset of mitral regurgitation [8]. In 1966, Barlow and Bosman reported on 4 patients presenting with late mitral murmurs and mid-systolic clicks in whom the whole posterior mitral leaflet protruded extensively into the left atrium during systole. They described the posterior mitral leaflet as “aneurysmal” or “billowing.” [9]. Also in 1966, Criley and colleagues performed cine-angiographic studies that showed late systolic motion of large mitral leaflets into the left atrium [5]. In 1968, mitral valve tissue was excised from 3 patients with myxomatous disease who underwent mitral valve replacement and was reported to show severe myxomatous degeneration of the leaflets and chordae [5]. Also in 1968, Ehlers and associates were the first to report left ventricular wall motion abnormalities in MMVD [10]. They observed a “systolic contraction ring” at the base of papillary muscles and hypothesized that this could lift both papillary muscles into the left ventricular cavity and towards the mitral annulus during systole, causing the chordae to slacken. In 1973, Liedtke and coworkers and Scampardonis and colleagues independently reported a high incidence of left ventricular “inflow wall” systolic motion abnormalities in the region of the base of papillary muscles [11, 12]. Liedtke and coworkers also noted that in patients with “mitral click syndrome” the papillary muscles moved toward the mitral valve annulus in systole, rather than towards the left ventricular apex [5].

## Methods

### Literature search and study selection

We conducted a systematic literature search using Pubmed and Embase, including references published up until September 2017. For this review, patients meeting the ‘surgical’ definition of Barlow’s disease i.e. a high degree of myxomatous degeneration with prolapse of one or both leaflets, were of interest. The following key words were screened: “Morbus Barlow”, “Barlow’s disease”, “genetic findings”, “genetic pathways”, “bileaflet prolapse”, “barlow mitral genetic”, “bileaflet mitral valve genetic analysis”, “myxomatous valve disease” and “myxomatous mitral valve”.

**Fig. 1:**
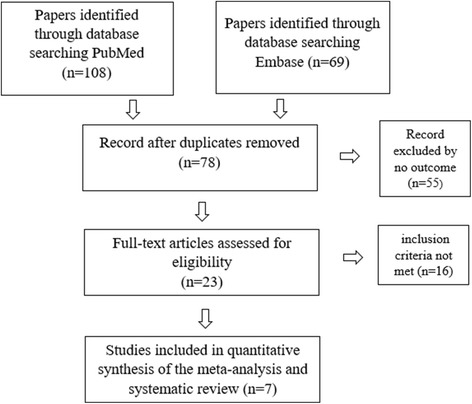
Trial enrollment showing the number of publications examining patients with myxomatous mitral valve disease who underwent genetic screening and echocardiography or surgery up to 09/2017. Patients with syndromic MVP were excluded. Thus, 7 papers were included in the further analysis

One reviewer performed the literature search. All relevant studies were included if published in a peer-reviewed journal and if the following inclusion criteria were met (Fig. 1):

- Affected individuals were diagnosed with myxomatous mitral valve disease and diagnosis was confirmed by histological examination or patients were found to have bileaflet mitral valve prolapse by echocardiography (i.e. involvement of the anterior and posterior leaflet)
- The patients had a non-syndromic MVP (mitral valve prolapse)
- Genetic analysis was performed

### Proband selection

All patients with known syndromic diseases were excluded from our review. Individuals with histological proof of MMVD who underwent mitral valve surgery and those with echocardiographic evidence of bileaflet prolapse were selected for subsequent genetic analysis, including their first-degree relatives. In the following paragraphs, we briefly describe patient’s selection process and methodology of the selected studies:

#### Chromosome 16 (MMVP1)

One recent study included families of 17 patients who underwent mitral valve repair and had histological verification of MMVD [13]. First degree relatives of these patients were screened using echocardiography in a double-blinded fashion to identify individuals with MMVP. The subjects were considered affected whenever the following criteria were met: (a) leaflet thickness > 5 mm, (b) a total leaflet displacement > 8 mm, and (c) late systolic mitral valve insufficiency. Genotyping was performed using 344 microsatellite markers from chromosomes 1 to 16.

#### Chromosome 13 (MMVP3)

Another study was carried out on one pedigree in a family of Western European descent [14]. The proband was a physician with diagnosed mitral valve prolapse. His family of 46 members of 3 living generations, were screened using echocardiography. “Classical” MVP was diagnosed if leaflet displacement exceeded 2 mm and maximal leaflet thickness was ≥ 5 mm. MVP was considered as “non-classical” if leaflet displacement exceeded 2 mm but maximal thickness was < 5 mm. Both echocardiographic variants were considered as MMVD positive. Genotypic analysis were performed using polymorphic microsatellite markers.

#### Chromosome 11 (MMVP2)

This analysis was performed using a single large pedigree of 41 individuals in 5 generations of a family of Western European descent [15]. The proband was identified as a volunteer during a teaching course on the topic of echocardiographic imaging. The subjects were classified as having MVP if leaflet displacement exceeded 2 mm. Patients were considered ‘indeterminate’ when leaflet displacement was ≤ 2 mm but there was evidence of either increased leaflet thickness, mitral regurgitation, left atrial enlargement, valve-related complications or disease progression over a period of 10 years. GeneHunter was used to perform genome scan. Multipoint parametric analysis was performed using 375 genetic markers and was confirmed by multipoint non-parametric analysis. Analysis was performed before the genome scanning to exclude coincident linkage to MMVP1 in Chr.16.

#### X-linked-MVD (XMVD)

A large family of French origin was analyzed [16]. The proband underwent aortic valve replacement due to severe aortic regurgitation caused by myxomatous aortic valve disease. His cousin had to undergo mitral valve surgery due to mitral valve dystrophy. Based on two affected patients, more than 300 family members were screened by echocardiography and a subsequent genetic study was performed using linkage analysis in 92 individuals [17, 18].

#### The role of TGF-β-signalling and increased oxidative stress in MMVD

Two studies analyzed 70 samples of mitral valve tissue which were collected during valve surgery [19, 20]. Half of these samples were acquired from patients who were diagnosed with MMVD by echocardiography and confirmed during surgery. The diagnosis of MMVP was verified by histopathological analysis of leaflet tissue. The remaining 35 control samples were collected from cardiac transplant recipients. Molecular changes in the mitral valves were assessed by means of qRT-PCR (quantitative real-time polymerase chain reaction), Western blotting and immunocytochemistry, and were compared between both subgroups [19, 20].

## Results

### Literature search and study characteristics

Based on our initial literature search, 172 potentially relevant references published between 1966 and 2016 were identified and 78 abstracts were screened. After exclusion of duplicate publications, a total of 160 citations were selected for full-text review. Full-text review revealed 23 potentially eligible studies. 7 of these met the necessary inclusion criteria and were selected for our cumulative analysis (Table 1).

**Table 1:** Gives an overview of study designs and methods

	Disse et al. [13]	Freed et al. [15]	Nesta et al. [14]	Monteleone et al. [18] Lardeux et al. [17] Kyndt et al. [16]	Hagler et al. [19]	Thalji et al. [20]
No° of pedigrees included	4	1	1	1	–	–
No° of individuals included	64	41	46	n/a (> 300)	48	22
Underlying disease	MMVD (in probands during surgery, in relatives by echo)	Mitral valve prolapse	Mitral valve prolapse	Co-existent myxomatous valvular dystrophy and hemophilia A	MMVD (during surgery)	MMVD (during surgery)
Exclusion criteria	n/a	(1) Marfan’s syndrome; (2) Genetical linkage to MMVP1	(1) Marfan’s syndrome /other CTD (2) genetical linkage to MMVP 1 or 2	(1) Marfan‘s and Ehlers-Danlos syndromes	(1) Marfan’s- syndome, Loeys-Dietz, osteogenesis imperfecta	n/a
Echochardiography	Transthoracic, blinded by two physicians	Transthoracic, blinded by two physicians	Transthoracic	Transthoracic, blinded by two physicians	n/a	–
Echocardiographic inclusion criteria	1)Thickness > 5 mm + leaflet displacement > 8 mm + annular displacement 2) Thickness 4-5 mm + leaflet displacement 3-8 mm + MVR	MVP > 2 mm	1)Classic: MVP > 2 mm + Leaflet thickening ≥ 5 mm 2) non-classic: MVP > 2 mm + leaflet thickness < 5 mm	Leaflet thickness > 4 mm	Severe mitral valve regurgitation	–
Genetic analysis + Gene expression	Genescan and Genotyper	Linkage and Genehunter, linkage to MMVP1 excluded	SLINK, linkage to MMVP1 + 2 excluded	Linkage analysis	TaqMan gene expression assay	qRT-PCR + Immunhistochemistry

No° of pedigrees and individuals tested are displayed. Underlying valve disease for each gene defect/ study is shown. Study design (e.g. double-blinded echocardiography, method used for genotyping) as well as in- and exclusion criteria are described

*m* male, *MVP* mitral valve prolapse, *n/a* not available, *f* female, *MVR* mitral valve regurgitation, *No*° number, *AML* anterior mitral leaflet, *MMVP* myxomatous mitral valve prolapse, *PML* posterior mitral leaflet, *qRT-PCR* quantitative real-time polymerase chain reaction

### Echocardiographical findings

**Fig. 2:**
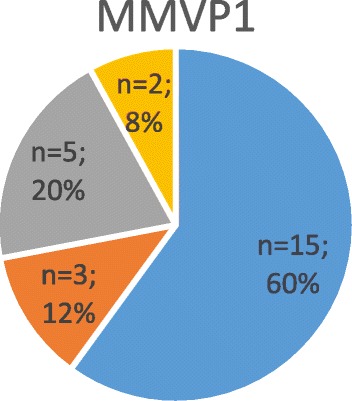
Shows the severity of mitral regurgitation in affected patients (i.e. positive genetic testing) with gene defect “MMVP1”

**Fig. 3:**
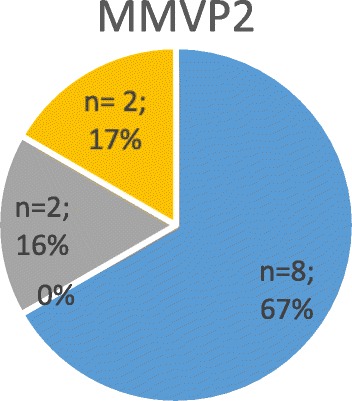
Shows the severity of mitral regurgitation in affected patients (i.e. positive genetic testing) with gene defect “MMVP 2”

**Fig. 4:**
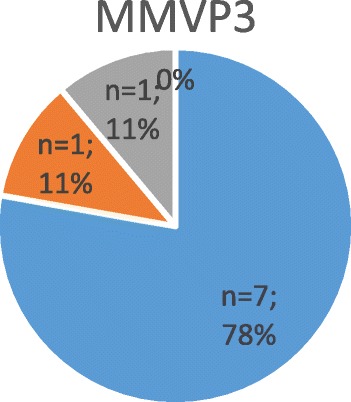
Shows the severity of mitral regurgitation in affected patients (i.e. positive genetic testing) with gene defect “MMVP 3”

**Fig. 5:**
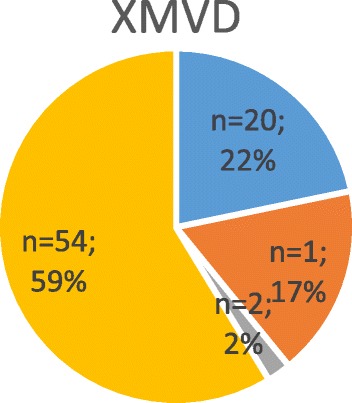
The pie chart shows grade of MR from the studies examining the X-linked mode of inheritance. The cumulative data of all 92 screened patients (i.e., echocardiography + genetic analysis) is displayed. Data of genetically affected patients is not provided

**Fig. 6:**
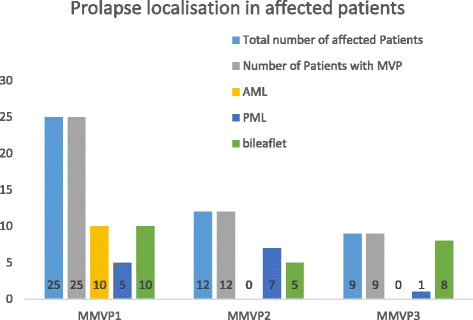
Prolapse localization of all affected patients is shown. Patients were considered to be affected either if surgery was performed due to valve regurgitation and myxomatous changes were confirmed by histological examination or if mitral valve prolapse with leaflet displacement of one or both leaflets ≥ 2 mm and leaflet thickening were present in echocardiography

**Fig. 7:**
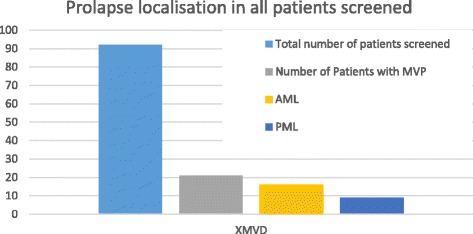
gives an overview of prolapse localization in all patients screened, including relatives of affected patients

Figures 2, 3, 4, 5, 6 and 7.

### Genetics of myxomatous mitral valve disease

Two loci of autosomal dominant inheritance causing bileaflet prolapse have been identified in chromosomes 16 and 13 [14, 15]. An autosomal dominant inheritance pattern for MVP was mapped to locus 11p15.4. Of note, the anatomical changes in these cases were not uniform, with 5 cases of bileaflet prolapse and 7 of isolated posterior leaflet prolapse [15]. Structural changes in filamin-A architecture caused by X-linked inheritance were found to cause myxomatous valvular dystrophy of the mitral and/or aortic valve combined with haemophilia A [16]. An overview of the findings of the identified studies is given in the following paragraphs.

#### Genetic findings in chromosome 16 (MMVP1)

Of the 17 families screened, 4 autosomal dominant pedigrees were identified [13]. Of these pedigrees, one was of Ashkenazi Jewish and the remaining three of French origin. Each pedigree had 5–9 affected relatives in 3–4 generations. Locus 16p12.1-p11.2 was identified in all affected individuals. 25 (32%) of the 79 family members screened using echocardiography were affected by MMVD causing MVP (Figs. 2 and 6).

#### Genetic findings in chromosome 13 (MMVP3)

A total of 43 family members were screened echocardiographically and genetically [14]. Nine (21%) patients met echocardiographic criteria of MVP, whilst 8 were diagnosed with bileaflet prolapse (Figs. 4 and 6). A total of 14 probands of this pedigree, including nine affected individuals, were screened genetically. The MMVP3 gene was mapped to the long arm of chromosome 13 and demonstrated an autosomal dominant inheritance pattern.

#### Genetic findings in chromosome 11 (MMVP2)

The pedigree analysis included 41 individuals in 5 generations of a family of Western European descent [15]. Echocardiograms and DNA were obtained from 28 (68%) subjects, of whom 12 individuals were diagnosed with MVP. Five individuals had a bileaflet prolapse, while the remaining subjects had an isolated posterior leaflet prolapse (Figs. 3 and 6). MMVP2 gene, which was revealed as a cause of mitral valve prolapse, was mapped to chromosome 11p15.4 and demonstrated an autosomal dominant inheritance pattern.

#### X-linked MVD (XMVD)

Two affected individuals who were scheduled for valve surgery due to valvular dystrophy were also found to suffer from a mild form of haemophilia A. A very thorough screening of the above mentioned family, including more than 300 individuals, revealed a total of 93 individuals with symptomatic myxomatous mitral and/or aortic valve disease. In all affected members the valvular disease was associated with a mild form of haemophilia A.

Moreover, we found an additional report on a patient undergoing surgery due to severe aortic valve regurgitation who was diagnosed with haemophilia A. This patient was also of French origin and had shared ancestry with the aforementioned family. A genealogical approach identified this ancestor as having lived in the eighteenth century, In this special family a total of 9 affected individuals with a co-existent valvular dystrophy and haemophilia A were identified [16, 18]. Using linkage analysis, the gene on chromosome Xq28 could be mapped. A positional cloning approach revealed a mutation in the *FLNA* gene in all affected family members. The X-linked change in filamin A was found to promote valvular dystrophy with an increase in anterior and posterior leaflet thickness and length in all mutation positive individuals. A total of 18 patients were found to have mitral valve regurgitation (Figs. 5 and 7). In affected male individuals, mitral valvular dystrophy was found to be more severe and was associated with a significant mitral annular enlargement when compared to their female counterparts (Table 2).

**Table 2:** Provides a detailed overview about known genetic findings in MMVD

	Disse et al. [13]	Freed et al. [15]	Nesta et al. [14]	Monteleone et al. [18]; Lardeux [17]; Kyndt [16]
Underlying disease	MMVD	MVP	MVP	Myxomatous valvular dystrophy + hemophilia type A
No° of included individuals (echocardiography + genetics)	64	28 (11 m, 17f)	43	92
Mean age (years)	49	34	55	32
Affected individuals (positive in genetic testing) (%)	25 (39%)	12 (43%)	9 (21%)	21 (23%)
MR present	23	10 (3 m,7f)	9 (5 m,4f)	38 (14 m, 24f)
- Trace to mild	15 (11 m, 4f)	8 (3 m, 5f)	7 (4 m, 3f)	20 (0 m, 20f)
- Moderate	3 (2 m, 1f)	0	1 (f)	16 (12 m, 4f)
- Severe	5 (3 m, 2f)	2 (0 m, 2f)	1 (m)	2 (2 m,0f)
Individuals with MVP	25 (39%) (16 m,9f)	12 (43%)	9 (21%)	21 (23%)
Prolapse localisation				
- AML	10	0	0	16 (12 m, 4f)
- PML	5	7 (3 m, 4f)	1(f)	9 (8 m, 1f)
- Bileaflet	10	5 (1 m, 4f)	8 (5 m, 3f)	n/a
Mean LVEF in MVP patients	n/a	65.5%	63.7%	69 + 8% m / 72 + 6% f
Chromosome	16	11	13	X
Locus	16p12.1.-p11.2	11p15.4	13.q31.3-q32.1	Xq28
Name of Gene	MMVP 1	MMVP 2	MMVP 3	XMVD
Mode of inheritance	Autosomal dominant	Autosomal dominant (incomplete penetrance)	Autosomal dominant	X-linked

It gives an overview of number of patients screened in each study, patients affected (i.e. positive genetic testing result), genetic mode of inheritance and gene locus. Grade of MR and localization of leaflet prolapse display phenotype and penetrance for each gene

*MR* mitral regurgitation, *m* male, *f* female, *MVP* mitral valve prolapse, *AML* anterior mitral leaflet, *PML* posterior mitral leaflet, *EF* ejection fraction, *No*° number, *n/a* not available

### Pathophysiological biomolecular pathways in MMVD

A recent study by Thalji and co-authors reported a differing gene expression patterns in myxomatous versus non-myxomatous mitral valves [20]. Transforming growth factor beta (TGF-β) signalling was found to be upregulated in myxomatous mitral valve tissue due to increased ligand expression and depression of canonical SMAD 2/3 signaling. Canonical bone morphogenetic protein (BMP) and Wnt/ ß-catenin signalling pathways were also found to be upregulated in MMVD and are associated with increased matrix remodelling, “pro-calcific” and pro-proliferative cellular responses. The expression of all 3 TGF-β subtype transcripts was significantly increased in the diseased mitral valve tissue and this was confirmed using immunoblotting [21].

Histopathological analysis demonstrated activated immune cells to be localised to the myxomatous mitral valve tissue [20]. Furthermore, oxidative stress was found to be significantly increased in the myxomatous mitral valves, induced by the stimulation of Nox 2 and Nox 4 gene expression (coding for NAPDH oxidase catalytic subunits) [19]. This pathway may further contribute to the amplification of TGF-ß signalling by SMAD 2/3 phosphorylation.

Recent in vitro studies using mitral leaflet specimens obtained by surgical excision have shown that myxomatous mitral valve changes might be pharmacologically modifiable by angiotensin receptor 2 blockers, a process which is based on TGF-ß modulation [21] (Table 3).

**Table 3:** Molecular pathways leading to MMVD discussed in the studies

	Hagler et al. [19]	Thalji et al. [20]	Geirsson et al. [21]
Underlying disease	Surgery due to myxomatous mitral valve disease, histological confirmation of myxomatous changes	Surgery due to myxomatous mitral valve disease	Surgery due to myxomatous mitral valve disease
No° of mitral valves	48	22	49
Individuals with myxomatous valves	24 (50%)	11 (50%)	26 (53%)
Echocardiographical findings	Severe mitral regurgitation: - 100% with MMVD vs. 58% with non-myxomatous valves	n/a	Severe mitral regurgitation in 26 diseased patients, not more than mild MVR in 23 non-diseased patients
Control group	Visually normal mitral valves from cardiac transplant recipients	Visually normal mitral valves from transplant patients	organ/tissue donors and explanted hearts of transplant recipients without abnormalities of mitral valve
Affected signalling protein	TGF-ß2-; BMP-; WNT/ß-catenin activation	TGF-ß, increased phosphorylation of SMAD2/3, increased oxidative stress	TGF-ß

Myxomatous valves were obtained through surgical excision of patients with myxomatous changes confirmed by histological examination. Cardiac transplant recipients with visually normal mitral valves formed the control groups. Five different signalling proteins as well as increased oxidative were found to play a role in development of MMVD

*No*° number, *MMVD* myxomatous mitral valve disease, *n/a* not available, *MVR* mitral valve regurgitation, *TGF-ß* transforming growth factor beta, *BMP* bone morphogenetic protein

## Discussion

The lack of a standardised definition of Barlow’s disease made this review a challenge. We focussed on patients with excessive myxomatous degeneration of the mitral valve, chordae elongation, annular dilatation and presence of prolapse of one or both leaflets of the mitral valve, as per the ‘surgical’ definition of Barlow’s syndrome. The aim of this review is to make physicians aware of MMVD as a heritable trait. Relatives of patients with surgically-confirmed Barlow’s disease may benefit from cardiological examination and follow-up. Our systematic literature review demonstrates genetic heterogeneity of MMVD with linkage to chromosomes 11, 13, 16 and the X chromosome. Moreover, the pathophysiological impact of increased transforming growth factor-ß expression and of downstream signalling proteins has been demonstrated [13–16, 19, 20]. An increase in oxidative stress also seems to be associated with MMVD [20].

The studies analysed varied significantly in terms of their size and design.

The three studies that analysed biomolecular mechanisms leading to MMVD have several limitations that should be highlighted. First, MMVP was only diagnosed histologically in one study [19]. Furthermore, the control group consisted of cardiac transplant recipients. Although their mitral valves showed no visual pathological changes, MMVD was not previously excluded [19, 20].

Myxomatous mitral valve disease appears to be the result of multiple genetic pathways.

## Conclusion

Large scale, multicentre segregation of MMVD patients and subsequent genome-wide association studies may allow the identification of additional relevant genes and further elucidation of pathways leading to the non-syndromic bileaflet mitral valve prolapse. Given the fact that MMVD affects young adult patients, identification of pathways leading to the development of diffuse myxomatous degeneration of leaflet tissue is of major clinical relevance. Early preventative interventions seeking to reduce the leaflet stress in genetically-susceptible individuals could potentially stop the progression of MMVD and prevent late valvular complications including mitral valve regurgitation, arrhythmias and endocarditis [15]. In vitro studies utilising surgically-excised mitral leaflet specimens have shown that myxomatous mitral valve changes might be pharmacologically modifiable by angiotensin receptor 2 blockers, a process which is based on modulation of TGF-ß [21]. This observation has potential clinical relevance in terms of the development of future pharmacological therapies for MMVD.

## Abbreviations

AML: Anterior mitral valve leaflet
BNP: Bone morphogenetic protein
Chr: Chromosome
ECM: Extracellular matrix
FED: Fibroelastic deficiency
FLNA-gene: Filamin A gene
MMVD: Myxomatous mitral valve disease
MMVP: Myxomatous mitral valve prolapse
MVP: Mitral valve prolapse
MVR: Mitral valve regurgitation
PML: Posterior mitral leaflet
qRT-PCR: Quantitative real-time polymerase chain reaction
TGF-ß: Transforming growth factor beta
